# Exposure to occupational antigens might predispose to IgG4‐related disease

**DOI:** 10.1002/hep.26999

**Published:** 2014-08-25

**Authors:** Lucas J. Maillette de Buy Wenniger, Emma L. Culver, Ulrich Beuers

**Affiliations:** ^1^ Department of Gastroenterology & Hepatology and Tytgat Institute for Liver and Intestinal Research, Academic Medical CentreUniversity of AmsterdamAmsterdamThe Netherlands; ^2^ Department of Gastroenterology & HepatologyOxford UniversityOxfordUK

**Abbreviations**
IAC: IgG4‐associated cholangitis
IgG4‐RD: IgG4‐related disease
PSC: primary sclerosing cholangitis

To the Editor:

IgG4‐associated cholangitis (IAC) and autoimmune pancreatitis are the predominant manifestations of IgG4‐related diseases (IgG4‐RD); their pathogenesis is enigmatic.^1^, ^2^ Serum IgG4 concentrations are generally elevated in IgG4‐RD but the biological implications thereof have not been explained.^3^ IgG4, traditionally regarded as a regulatory antibody, has been consistently reported to be upregulated in chronic immune stimulation, as illustrated by an elevation of bee‐venom‐specific IgG4 in beekeepers.^4^ As reported in this journal, we recently found clonal expansions of IgG4‐switched B‐cells in patients with IAC, using a novel next‐generation sequencing protocol.^5^ That led us to speculate that chronic antigenic stimulation underlies the often tremendously elevated levels of serum IgG4 in IgG4‐RD. As it was our clinical impression that blue‐collar workers were dominating our cohort of IAC patients, we hypothesized that chronic occupational antigen exposure could play a crucial pathogenetic role for the mainly elderly male IgG4‐RD patients.

Using a questionnaire, we carefully investigated the job history of our mostly retired IgG4‐RD patients. Of 25 patients with IAC and/or autoimmune pancreatitis, 88% had a history of blue‐collar work of at least 1 year, but often of a whole career (Table 1: e.g., building contractors, plumbers), much more than could be expected on the basis of historical Dutch occupational records. Chronic exposure to solvents, industrial and metal dusts, and pigments and oils used in the automotive industry were among the most frequent potential occupational hazards. In comparison, among a disease control cohort of 21 patients with primary sclerosing cholangitis (PSC), a male‐predominant disease with similar clinical characteristics, only 14% reported a history of working in a blue‐collar profession.

**Table 1:** Job History and Occupational Exposures of the Amsterdam Cohort of Patients With IgG4‐RD (IgG4‐Associated Cholangitis, Autoimmune Pancreatitis)

Job history of 25 patients from the Amsterdam cohort (> 1 year)	Recalled regular occupational exposures (> 1 year)
1. Musician, painter, metal worker, carpenter	solvents, car paint, metal, pigments
2. Carpenter	solvents, sawdust, wood, chipboard
3. Glass worker, project manager at multinational	glass dust, glass components, lead, barium, cobalt, nickel, lead, silica, industrial dust, building sites
4. Plasterer	solvents, chalk dust, sawdust, wood, chipwood
5. Industrial warehouse forklift driver	unknown (deceased)
6. Industrial fuel/waste oil laboratory, skipper	solvents, crude oil, ship waste oil, chemicals
7. Miner, tiler, bath superintendent	solvents, silica dust, mine dust, asbestos, glue
8. Metal worker, textile worker	solvents, metal dust, textiles, pigments, paints
9. Shipping	solvents, asbestos, crude oil
10. Painter, army officer, flight arrangements, tomato farmer	solvents, paint, pigments, kerosene, pesticides, friction plate dust
11. Painter	solvents, paint, pigments, dust
12. Small machine factory owner	solvents, car paint, metal dust, asbestos, oils
13. Builder, plumber	plumbing materials, dust, sawdust, glue, lead
14. Self‐employed optometrist	lense glass dust, lense plastic dust, acetone
15. Carpenter	solvents, sawdust, clipboard, glue
16. Bricklayer, industrial cleaner of house walls	solvents, silica dust, concrete dust, brick dust, asbestos
17. Mud worker, shipping, mud industry manager	solvents, oil products, dust
18. Builder, painter	solvents, sawdust, clipboard, paints
19. Car industry worker	solvents, oil products
20. Historian, rebuilt 3 houses during last 20 years	solvents, sawdust, silica dust, paint
21. Builder, wall miller	solvents, sawdust, silica dust, dust
22. Hospital cleaner	cleaning products
23. Teacher	no known exposures
24. Nurse	no known exposures
25. Unknown (deceased)	unknown (deceased)

Using the same questionnaire, a trial nurse blinded to our hypothesis and the Amsterdam results replicated this investigation among the Oxford cohort of 44 patients with established IgG4‐RD. It was found that 61% of the patients had blue‐collar professions and recalled chronic exposures to potentially harmful compounds. Again, intensive and prolonged exposure to solvents, industrial dusts, pesticides, or industrial oils or polymers was reported by 52% of IgG4‐RD patients. In a control cohort of 27 PSC patients from Oxford with elevated serum IgG4 (>1.4 g/L) and no histological evidence of IAC, the percentage of blue‐collar workers was 22%. Among the PSC patients, 7% reported any (often incidental) exposure to these compounds.

Our earlier finding of clonal expansions of IgG4‐switched B cells in patients with IgG4‐RD^5^ is compatible with the presence of an antigen‐driven immune process in these individuals. Given our observed high rate of chronic occupational exposure of two independent cohorts of IgG4‐RD patients suggests that chronic exposure to occupational antigens may play a role in the initiation and/or maintenance of IgG4‐RD in susceptible individuals.


Lucas J. Maillette de Buy Wenniger, M.D.^1^*
Emma L. Culver, M.D.^2^*
Ulrich Beuers, M.D.^1^

^1^Department of Gastroenterology and Hepatology
Tytgat Institute for Liver and Intestinal Research
Academic Medical Center, University of Amsterdam
Amsterdam, The Netherlands
^2^Department of Gastroenterology and Hepatology
Oxford University
Oxford, UK
